# Strain Elastography as a Valuable Diagnosis Tool in Intermediate Cytology (Bethesda III) Thyroid Nodules

**DOI:** 10.3390/diagnostics9030119

**Published:** 2019-09-13

**Authors:** Dana Stoian, Florin Borcan, Izabella Petre, Ioana Mozos, Flore Varcus, Viviana Ivan, Andreea Cioca, Adrian Apostol, Cristina Adriana Dehelean

**Affiliations:** 1 “Victor Babes” University of Medicine and Pharmacy Timisoara, Internal Medicine 2nd Department, 2nd Eftimie Murgu Square, 300041 Timisoara, Romania; stoian.dana@umft.ro (D.S.); ivanmvivi@yahoo.com (V.I.); 2 “Victor Babes” University of Medicine and Pharmacy Timisoara, Analytical Chemistry Department, 2nd Eftimie Murgu Square, 300041 Timisoara, Romania; 3 “Victor Babes” University of Medicine and Pharmacy Timisoara, Obstetrics-Gynecology Department, 2nd Eftimie Murgu Square, 300041 Timisoara, Romania; petre.izabella@umft.ro; 4 “Victor Babes” University of Medicine and Pharmacy Timisoara, Physiopathology Department, 2nd Eftimie Murgu Square, 300041 Timisoara, Romania; 5 “Victor Babes” University of Medicine and Pharmacy Timisoara, Surgery 2nd Department, 2nd Eftimie Murgu Square, 300041 Timisoara, Romania; varcus.florian@yahoo.com; 6 CFR Clinical Hospital, Pathology Department., 13–15th Tudor Vladimirescu Street, 300173 Timisoara, Romania; cioca.andreea19@gmail.com; 7 “Pius Branzeu” County Emergency Hospital Timisoara, 2nd Eftimie Murgu Square, 300041 Timisoara, Romania; apostolvadrian@gmail.com; 8 “Victor Babes” University of Medicine and Pharmacy Timisoara, Toxicology Department, 2nd Eftimie Murgu Square, 300041 Timisoara, Romania; cadehelean@umft.ro

**Keywords:** Bethesda III category, cancer risk, modified TI-RADS, strain elastography

## Abstract

Fine needle aspiration (FNA) is considered the gold standard in the diagnostic of thyroid nodules. Using the recommended BETHESDA reporting system, up to 20% of results are classified as intermediate cytology. As there is no consensus whether ultrasound evaluation, lobectomy or surgery is the best treatment option, intermediate cytology results are considered a grey zone of the FNA. The main aim of our study was to evaluate the performance of combined advanced ultrasound techniques in the process of diagnosis and evaluation of the intermediate cytology cases after FNA. We evaluated 54 consecutive cases with intermediate cytology on FNA, using conventional B-mode ultrasound (2B), and strain elastography, using a linear multifrequency 6–13 MHz linear probe (Hitachi Prerius Machine, Hitachi Inc, Japan). All nodules were classified with our Thyroid Imaging Report and Data System (TI-RADS) proposed model, considering: vertical appearance, with antero-posterior diameter bigger than the transvers diameter, the so called taller than wide shape, irregular borders, intranodular inhomogeneity, marked hypoecogenicity, micro calcifications, the presence of suspect lymph nodes, and increased stiffness as suspicious for malignancy. The classification outcomes were compared with the pathology results, considered the gold standard diagnosis. The prevalence of cancer was 28.8%, with 13/45 cases having a clear diagnostic of cancer. Six cases were diagnosed with borderline follicular neoplasia, a category with unclear evolution, also considered as malignant in the analysis of the imaging results. In total, 16/19 cancer cases had increased stiffness on elastography. The cancer prevalence increased with TI-RADS category, being 25% in TI-RADS 4b category and 92.8% in TI-RADS 5 category. The AUROC (Area Under Receiver Operating Curve) of elastography alone, in differentiation of malignant thyroid nodules was 74.9%; the combination of elastographic and conventional ultrasound characteristics generated an even better AUROC, of 84.5%. The combined conventional ultrasound and elastography identified thyroid cancer in cases with intermediate cytology with a sensitivity of 89.5% with a specificity of 50%. High risk thyroid nodules, identified by combined high risk conventional ultrasound characteristics and increased stiffness, on strain elastography, are highly predictive for malignancy, in the intermediate cytology cases.

## 1. Introduction

Thyroid nodules are very common in the population and most of them are benign. High-resolution ultrasound (US) is the method of choice for the investigation of thyroid nodules, allowing the selection of the cases that need to be punctured [1]. Fine needle aspiration (FNA) has a pivotal role in pre-surgical selection of the patients as it is the best procedure for differentiating benign from malignant nodules, and is considered the gold standard in the diagnosis of thyroid malignancy [2,3]. However, the intermediate risk category, with undetermined results is still an unclear category [1,3,4,5]. The management of the patients with indeterminate cytology is a great challenge for the clinicians [6], active follow up, radical or diagnostic surgery being recommended [2,3]. The problem of this special diagnostic category is a relatively wide range of cancer risk, mean of 16%, comprised between 6–48% of cases [7,8,9].

Elastography is a dynamic technique that uses ultrasound to estimate the tissues’ stiffness and elasticity. When used in combination with conventional, grey scale ultrasound, elastography highlights the fine details of the thyroid nodules, and it is also helpful in characterization of other structures as cervical lymph nodes, post-thyroidectomy residual tissue or thyroiditis pseudo-nodules [10,11]. Currently, there are many elastographic techniques used in clinical practice: shear wave elastography biplane method, point share wave elastography, strain/real-time elastography, respectively fibroscan. [12]. The method is non-invasive, completely painless for the patient and can be easily performed even during routine ultrasound examinations.

A significant body of evidence recognizes the diagnostic quality of strain elastography, with sensitivity and specificity ranging between 65–100% and 44–96%, respectively, and an overall accuracy of 85–95% [13,14,15,16,17]. Moreover, the sensitivity and specificity increased when elastography was added as a parameter of The Thyroid Imaging Reporting and Data System (TI-RADS) [16,18]. However, the role of elastography in the management of nodules with indeterminate cytology is still debated since the results of the studies are contradictory. There are many positive studies, showing that increased stiffness is associated with thyroid cancer, and low stiffness is highly characteristic of a benign lesion [19,20] According to these data, we are considering including elastography parameters along with the conventional ultrasound parameters incorporated in the standard TI-RADS model, as this could increase the sensitivity and specificity of the standard TI-RADS.

Thus, the aim of the present study was to determine the diagnostic value of elastography both alone and when combined with the Russ TI-RADS model [18], compared with the standard TI-RADS model, for the assessment of the special category of thyroid nodules with indeterminate cytology.

## 2. Results

In this study 45 patients were enrolled (38 females and 7 males), aged between 21 and 82 years (mean 44.64 years). Following surgery, 13 (28.8%) patients got the final diagnosis of malignancy: one case (7.7%) had follicular carcinoma and 12 cases (92.3%) had papillary carcinoma. Thirty-two patients (71.1 %) had non-neoplasic lesions including 14 (43.75 %) follicular adenomas, 8 (25%) nodular goiter/hyperplasic nodules, 6 follicular neoplasia with unclear risk (18.75%), 3 Hurthle cells adenoma, (9,3%) one granulomatous thyroiditis (3.1%) and one (3.1%) Hashimoto thyroiditis.

Special attention was needed for the so called “follicular borderline lesions” that include those with invasion into the capsule beyond the bulk of the lesion without affecting the full thickness of the capsule or situations in which islands of tumor are trapped within a capsule, associated with perpendicular rupture of collagen [21].

As follicular neoplasia “with unclear risk” is a borderline lesion that needs watchful waiting [22,23,24,25], due to uncertain risk and evolution, we considered it malignant, even if postsurgical radioactive iodine treatment was not required in all cases.

Malignant nodules showed a median strain ratio value of 5.50, significantly (*p* = 0.0003) higher compared with the 3.88 median value noted in the benign cases.

The diameter of the nodules varied between 8.7 mm and 43.6 mm with a mean of 18.9 mm. The benign nodules were larger in size compared with malignant ones (mean size 18.1 mm versus 14.4 mm).

At elastography, 7 nodules had a score of 1, 8 nodules had a score of 2, 15 nodules had a score of 3 and 13 nodules had a score of 4. In the stiffness score 1 and 2 cases, the “positive cases” were all borderline stiffness neoplasia, which, because of their unclear prognostic, were considered cancer lesions. The vast majority of score 4 cases, 73.3% were cancer cases. One third of score 3 cases were cancers. Considering scores 1 and 2 as probably benign and scores 3 and 4 as suspicions for malignancy, elastography showed 84.21% sensibility, 53,84% specificity, positive predictive value 57.14% and negative predictive value 82.35%. A higher risk of malignancy was noted in rigid nodules (*p* = 0.020). The data are depicted in Table 1.

Based on combined TI-RADS system, 5 nodules had a score of 3, 10 nodules had a score of 4a, 16 nodules had a score of 4b and 14 nodules had a score of 5. Considering scores 3 and 4a as benign and scores 4b and 5 as suspicions for malignancy, the model showed 89.5% sensibility, 50% specificity, positive predictive value 56.6% and negative predictive value 86.6%. The data are depicted in Table 2.

The vast majority of cancer cases scored high in stiffness index and combined TI-RADS score.

**Figure 1:**
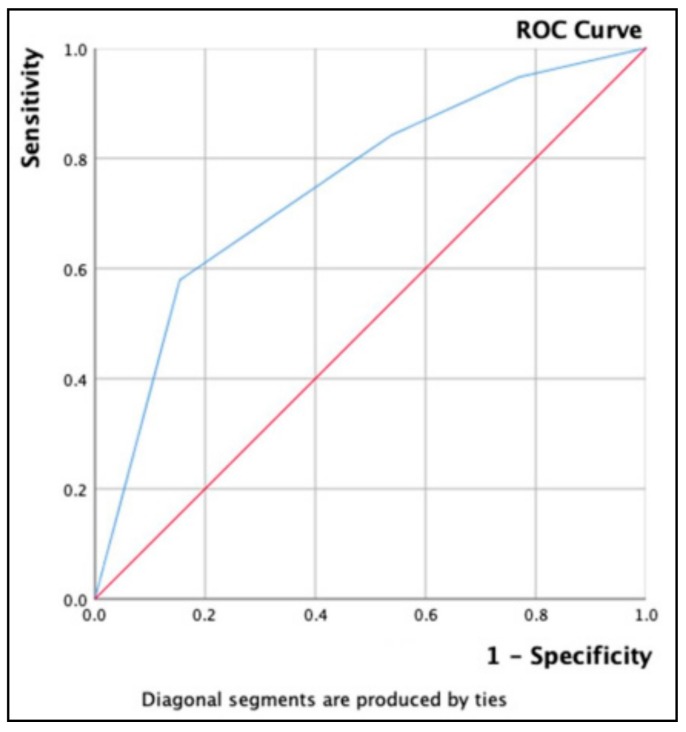
AUROC (Area Under Receiver Operating Curve) for stiffness index (elastography only evaluation) in intermediate cytology cases: (AUS/FLUS (Atypia of undetermined significance/ Follicular lesion of undetermined significance) cases)—45 patients.

As seen in Figure 1, the area under ROC curve, was excellent for elastography only information, of 74.9%, 95% CI = 0.602–0.896.

**Figure 2:**
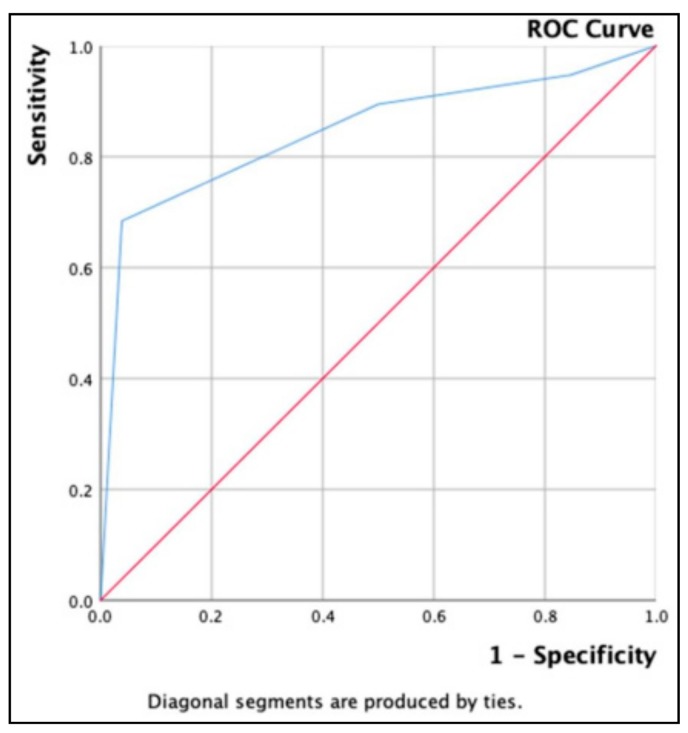
AUROC for combined TI-RADS in AUS/FLUS cases—45 patients.

Figure 2 is presenting the AUROC for combined Thyroid Imaging Report and Data System (TI-RADS) model, equaling 84.5%, 95% CI = 0.718–0.974.

The diagnostic quality of combined TI-RADS system, in the diagnostic thyroid neoplasia, in the 7 cases with FN on cytology report, showed a sensitivity of 100% but with a low specificity of 33.3%, with a Positive Predictive Value (PPV) of 66.6% but with Negative Predictive Value (NPV) of 100%. Elastography only described a sensitivity of 100%, with a better specificity of 66.6%, PPV = 80% with NPV = 100%.

## 3. Discussion

The main goal of the thyroid nodules’ evaluation is to exactly determine whether they are benign or malignant in order to reduce the number of unneeded FNAC and surgeries. FNAC is the most accurate tool for characterizing thyroid nodules, however, the main diagnostic limitation is represented by indeterminate lesions, representing 10–25% of all cytological results [26]. More than half of the lesions with indeterminate cytology proved to be histologically benign, thus finding new tools for differentiation of benign from malignant thyroid nodules is imperative. Over time, different diagnostic procedures have been proposed to overcome the diagnostic limit of the FNAC, however, the results were modest. According to The American Association of Clinical Endocrinology (AACE) and The American Thyroid Association (ATA), the current management of intermediate FNAC lesions is to evaluate each case individually according to the ultrasound risk category of the nodule and the presence of suggestive clinical and especially, historical/anamnestic risk factors [3,4]. Still, there is a wide spectrum of accepted recommendations: active follow-up, diagnostic surgery or total thyroidectomy [3,4].

In our study, in the 45 cases with AUS/FLUS cytology report, the percentage of malignant nodules was 42.2%, higher than in previous reported series regarding indeterminate lesions [17,19,27,28,29], but lower than other described prevalence [3,30,31]. Most of the lesions were papillary carcinomas (63.15%), while only one case was diagnosed as follicular carcinoma (5.2%), and 6 cases were evaluated as borderline follicular neoplasia (31.6%). These 6 cases were responsible for the high cancer prevalence in our study. If these cases had not been considered potentially malign, due to the uncertain future evolution and treatment [21,22,23,24,25], the clear cancer cases prevalence would be just 28.9%.

A high percentage of papillary carcinomas was also reported by Trimboli et al. who found 14 papillary carcinomas, one follicular carcinoma and one medullary carcinoma in a series of 42 nodules with indeterminate significance [32]. In addition, Lippolis et al. reported on 34 papillary carcinomas among 36 malignant nodules [33].

Due to its high sensitivity, US (Ultrasound) is the first method of choice for the initial evaluation of a thyroid nodule. Irregular margin, calcifications, hypoechogenicity, a solid composition and a taller than wide shape are parameters that are highly suspicious for malignancy [34]. All these parameters are included in the standard TI-RADS model and also in Russ’ TI-RADS model. However, calcifications are rarely found in carcinomas and hypoechogenicity and solid composition can be frequent found in benign nodules, thus conventional US has a low accuracy in differentiating malignant nodules from benign ones especially when only a single US parameter is used [19].

As a rigid nodule should always be suspicious for malignancy, elastography, a technique that assesses tissue stiffness, has received great interest [13]. The results are encouraging, even from a small series of indeterminate thyroid nodules [35,36,37,38]. However, the results of Lippolis et al. were contradictory [33]. Our study included 45 patients with indeterminate cytology and we found that elasticity scores were higher in malignant nodules compared with benign ones. Although the sensitivity of the procedure was high (84.21%), the specificity was only 53.84%. Hurthle cell adenoma and follicular adenoma were responsible for the benign cases with increased stiffness.

There is evidence that a scoring system that combines different techniques may offer a better evaluation of the thyroid nodules. TI-RADS system, derived from the breast imaging reporting and data system (BI-RADS), was developed to stratify the risk of malignancy by using ultrasound. Russ was the first that included elastography as a parameter in TI-RADS system and found that the specificity and sensitivity were improved when elastography was used in combination with gray-scale US than elastography alone [18]. In the present study, the specificity remained the same but the sensitivity increased to 89.47% when elastography was considered, despite the grey scale ultrasound parameters. We noted an increased risk of malignancy with increasing the TI-RADS categories 89.5% of all pathologically confirmed malignant nodules had a TI-RADS score of 4b or 5. However, there were 13/26 false positives among those that scored 4b or 5 in TI-RADS system, results that are in line with those of Xue et al. [39]. The negative predictive value of both elastography (82.3%) and TI-RADS model (86.6%) increases the confidence of recommending active follow up in intermediate cytology cases with low risk ultrasound and elastography appearance, as described in other studies [19,20]. As Doppler information is currently not used in the standard TI-RADS [18] we did not use them in our analysis.

The diagnostic performance in follicular neoplasia cases is similar, but the total number of cases is small, so no conclusions can be formulated in this special group of intermediate cytology cases.

## 4. Materials and Methods

### 4.1. Patients

The study included 45 consecutive patients with intermediate cytology (Bethesda III category) examined in our Endocrine Unit between January 2018 and December 2018. They were recruited from the 237 patients which performed FNA in the study period. Surgical treatment (lobectomy or total thyroidectomy) was performed in all cases, with pathology report being the gold standard for the final diagnostic. A complete US evaluation was performed prior FNA by an operator with more than 10 years’ experience. This included conventional gray scale and strain elastography. The study was performed in accordance with the ethical guidelines of the Helsinki Declaration and was approved by the Ethics Committee of our Center. Written informed consent was obtained from all patients prior to inclusion.

### 4.2. Ultrasound Evaluation

Ultrasound evaluation was performed with a Hitachi Preirus device (Hitachi Medical Corporation, Tokyo, Japan) with 6–13 MHz linear probe. SE (Strain/real-time elastography) was performed in accordance with Rago et al. [40] recommendations using mild external pressure, applied by the technician, checked on the pressure scale. The length and intensity of pressure was based on the stability of the images, and the correct pressure impact of evaluation was according to the stability of the images. The pressure scale is recorded for each case, as pressure scale 3 or 4 (highlighted in green on the left side of the screen, or as oscillations between +/−1 standard deviation, at the bottom of the screen). The pressure scale is a nonadjustable preset of the ultrasound device. The Region of Interest (ROI) has to be big enough to comprise the sufficient thyroid tissue surrounding the nodular lesion. Real time evaluation was used. Only stable images, in the color map schema, with proper pressure were recorded.

The qualitative SE (standard blue red green color map) was determined for each nodule. The strain ratio was obtained by comparing the stiffness of the nodule with the surrounding healthy, non-nodular tissue. Firstly, we defined the nodular margins, as the first circle, and we defined the second circle at the same level (depth), in the healthy thyroid tissue, surrounding the nodule. The US machine calculated the strain ratio.

**Figure 3:**
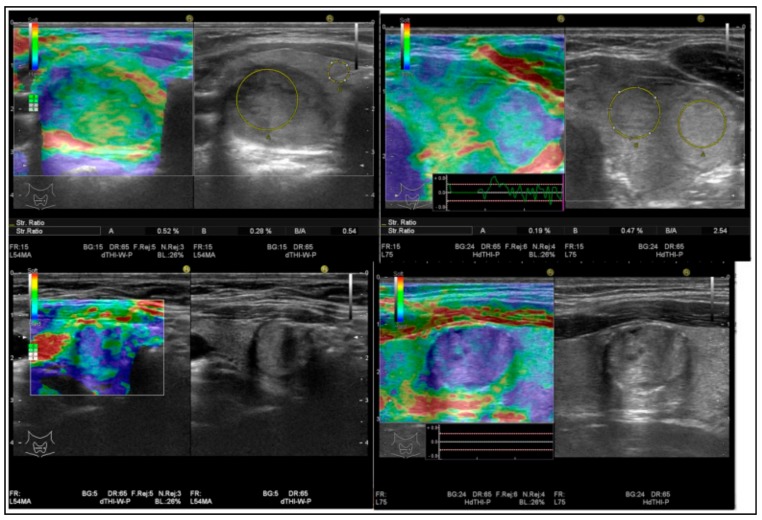
Intermediate cytology nodules of fine needle aspiration (FNA), with different stiffness degrees: low (score 1 = upper left)–intermediate (score 2 = upper right)–hard (score 3 =lower left)–very hard (score = 4 lower right).

Figure 3 shows the stiffness score categories used in our study.

The nodules were classified using the modified TI-RADS system described by Russ et al. [18] (Table 3). The following characteristics were considered as suspicious: taller than wide, irregular borders, intranodular inhomogeneity, marked hypoecogenicity, micro calcifications, presence of suspect lymph nodes, and increased stiffness. Increased stiffness is defined as color map code 3 and 4 (on qualitative SE) respectively a strain ratio was equal or higher than 4 (in semi-quantitative SE) [16]. The threshold value of 4 is the value that our center identified as suggestive, in previous studies. There are different thresholds defined for different elastographic devices [13,14,17].

### 4.3. FNA

All FNA were performed under US guidance by an experienced endocrinologist with over 20 years of experience in our department. The patients were placed in a supine position, with a pillow placed under the patient’s shoulders to ensure a slight neck hyperextension. The overlying skin was sterilized using an alcohol pad and local anesthesia was performed with 1–2 mL Lidocaine hydrochloride solution injected into the skin and superficial subcutaneous tissue at the predetermined site. A high resolution (7.5–15 MHz) linear-array transducer with a sterile cover was used to locate the lesion. Prior to the FNA color Doppler mapping was performed to identify large blood vessels in and around the nodule that were avoided to prevent vascular injury. The maneuver employed 25 and 27 gauge sterile needles attached to 10 cc syringes. The needle was inserted parallel to the transducer, with direct visualization and confirmation that the needle has reached the lesion. The needle performed “coring” movements through which the material was collected. We utilized two passes/nodules and the sampled cellular material was placed on glass slides for conventional smears. A minimum of 5 slides/case were obtained. The slides were quickly fixed in 95% ethyl alcohol for the Papanicolaou stain [41]. Specimen adequacy was not evaluated on site. The slides were assessed, reviewed and reported by a cytopathologist with experience in thyroid pathology. The adequacy assessment and reporting were made using the Bethesda System for Reporting Thyroid Cytopathology.

### 4.4. Surgical Intervention and Pathological Examination

The patients underwent unilateral lobectomy or total thyroidectomy with lymph node dissection in few selected cases. The procedures were performed by the surgeons in our team while the histopathological diagnosis was made in the Pathology Department by thyroid pathology specialists. When needed, immunohistochemical reactions for HBME, CK-19, TTF-1 and Ki-67 were used.

### 4.5. Statistical Analysis

SPSS statistical software version 17 (SPSS Inc, Chicago IL, USA) was used for the descriptive analysis of all the parameters which includes the biodata, results of the FNAB as well as the elastography. Cross tabulation was used in analysis of association between two or more parameters. Correlational analysis was also used to show association as well as relationship between them to discover if dependency or independency can be seen. Chi square as well as ANOVA were also used for inferential statistics. SPSS statistic was also used to draw the ROC curve for strain ratio in order to determine the cutoff point for determination of malignancy and benignity of the tumor.

## 5. Conclusions

In conclusion, our results suggest that including elastography as a parameter of TI-RADS score increases the accuracy of diagnostic of the patients with indeterminate thyroid cytology and may lead to a reduction in diagnostic surgeries, in cases with low stiffness. However, the system has some drawbacks: a large number of benign cases being stiff on elastographic evaluation, such as Hurthle cell neoplasia and follicular adenoma. The question about borderline follicular neoplasia remains open.

## Figures and Tables

**Table 1:** Prevalence of neoplastic cases according to the reported stiffness.

Stiffness Score	Prevalence of Cancer (%)
1	1/7 (14.3%)
2	2/8 (25.0%)
3	5/15 (33.3%)
4	11/15 (73.3%)

**Table 2:** Prevalence of neoplasia in thyroid nodules according to the risk score.

TI-RADS Score	Prevalence of Cancer (%)
3	1/5 (20.0%)
4a	1/10 (10.0%)
4b	4/16 (25.0%)
5	13/14 (92.8%)

**Table 3:** Modified TI-RADS classification (adapted from Russ et al. [18]).

TI-RADS	Interpretation	Ultrasonographyc Finding
1	Normal thyroid	Normal thyroid tissue without any nodular aspect
2	Constantly benign aspect	Simple cyst, spongiform nodule, “white knight”, isolated macro calcification, nodular hyperplasia
3	Very probable benign	No signs of high suspicion, isoechoic or hyperechoic
4a	Undetermined	No signs of high suspicion, mildly hypoechoic, encapsulated
4b	Suspicious	1 or 2 signs of suspicion
5	Highly suspicious	>3 sign of suspicion

## Abbreviations

AACE	American Association of Clinical Endocrinology
ATA	American Thyroid Association
AUROC	Area Under Receiver Operating Curve
AUS	Atypia of undetermined significance
BI-RADS	Breast Imaging Reporting and Data System
cc	centimeter cube
CI	Confidence Interval
FLUS	Follicular lesion of undetermined significance
FNA	Fine Needle Aspiration
FNAB	Fine Needle Aspiration Biopsy
FNAC	Fine Needle Aspiration Cytology
NPV	Negative Predictive Values
PPV	Positive Predictive Values
ROC	Receiver Operating Curve
SE	Strain/real-time elastography
TI-RADS	Thyroid Imaging Reporting and Data System
US	Ultrasound

## References

[1] Hoang J.K., Lee W.K., Lee M., Johnson D., Farrell S. (2007). US features of thyroid malignancy: Pearls and pitfalls. Radiographics.

[2] Gharib H., Papini E., Garber J.R., Duick D.S., Harrell R.M., Hegedüs L., Paschke R., Valcavi R., Vitti P. (2016). AACE/ACE/AME Task Force on Thyroid Nodules. American Association of Clinical Endocrinologists, American College of Endocrinology, and Associazione Medici Endocrinologi medical guidelines for clinical practice for the diagnosis and management of thyroid nodules – 2016 update. Endocr. Pr..

[3] Haugen B.R., Alexander E.K., Bible K.C., Doherty G.M., Mandel S.J., Nikiforov Y.E., Pacini F., Randolph G.W., Sawka A.M., Schlumberger M. (2016). 2015 American Thyroid Association Management Guidelines for Adult Patients with Thyroid Nodules and Differentiated Thyroid Cancer: The American Thyroid Association Guidelines Task Force on Thyroid Nodules and Differentiated Thyroid Cancer. Thyroid.

[4] Giusti M., Massa B., Balestra M., Calamaro P., Gay S., Schiaffino S., Turtulici G., Zupo S., Monti E., Ansaldo G. (2017). Retrospective cytological evaluation of indeterminate thyroid nodules according to the British Thyroid Association 2014 classification and comparison of clinical evaluation and outcomes. J. Zhejiang Univ. Sci. B.

[5] Cibas E.S., Ali S.Z. (2017). The 2017 Bethesda system for reporting thyroid cytopathology. Thyroid.

[6] Valderrabano P., McIver B. (2017). Evaluation and management of indeterminate thyroid nodules: The revolution of risk stratification beyond cytological diagnosis. Cancer Control..

[7] Bongiovanni M., Spitale A., Faquin W.C., Mazzucchelli L., Baloch Z.W. (2012). The Bethesda System for Reporting Thyroid Cytopathology: A meta-analysis. Acta Cytol..

[8] Ohori N.P., Nikiforova M.N., Schoedel K.E., LeBeau S.O., Hodak S.P., Seethala R.R., Carty S.E., Ogilvie J.B., Yip L., Nikiforov Y.E. (2010). Contrbution of molecular testing to thyroid fine needle aspiration cytology of “follicular lesion of undeterminated significance/atypia of undeterminated significance”. Cancer Cytopathol..

[9] Bongiovanni M., Crippa S., Baloch Z., Piana S., Spitale A., Pagni F., Mazzucchelli L., Di Bella C., Faquin W. (2012). Comparison of 5-tiered and 6-tiered diagnostic systems for the reporting of thyroid cytopathology: A multi-institutional study. Cancer Cytopathol..

[10] Bhatia K.S.S., Cho C.C.M., Tong C.S.L., Yuen E.H.Y., Ahuja A.T. (2012). Shear wave elasticity imaging of cervical lymph nodes. Ultrasound Med. Biol..

[11] Monpeyssen H., Tramalloni J., Poirée S., Hélénon O., Correas J.M. (2013). Elastography of the thyroid. Diagn. Interv. Imaging.

[12] Gay S., Schiaffino S., Santamorena G., Massa B., Ansaldo G., Turtulici G., Giusti M., Thyroid Team At The Policlinico San Martino Genoa (2018). Role of Strain Elastography and Shear-Wave Elastography in a Multiparametric Clinical Approach to Indeterminate Cytology Thyroid Nodules. Med. Sci. Monit..

[13] Bojunga J., Herrmann E., Meyer G., Weber S., Zeuzem S., Friedrich-Rust M. (2010). Real-time elastography for the differentiation of benign and malignant thyroid nodules: A meta-analysis. Thyroid.

[14] Razavi S.A., Hadduck T.A., Sadigh G., Dwamena B.A. (2013). Comparative effectiveness of elastographic and B-mode ultrasound criteria for diagnostic discrimination of thyroid nodules: A meta-analysis. Ajr Am. J. Roentgenol..

[15] Colakoglu B., Yildirim D., Alis D., Ucar G., Samanci C., Ustabasioglu F.E., Bakir A., Ulusoy O.L. (2016). Elastography in Distinguishing Benign from Malignant Thyroid Nodules. J. Clin. Imaging Sci..

[16] Stoian D., Timar B., Derban M., Pantea S., Varcus F., Craina M., Craciunescu M. (2015). Thyroid Imaging Reporting and Data System (TI-RADS): The impact of Quantitative Strain Elastography for better stratification of cancer risks. Med. Ultrason..

[17] Cantisani V., Grazhdani H., Drakonaki E., D’Andrea V., Di Segni M., Kaleshi E., Calliada F., Catalano C., Redler A., Brunese L. (2015). Strain US Elastography for the Characterization of Thyroid Nodules: Advantages and Limitation. Int. J. Endocrinol..

[18] Russ G., Royer B., Bigorgne C., Rouxel A., Bienvenu-Perrard M., Leenhardt L. (2013). Prospective evaluation of thyroid imaging reporting and data system on 4550 nodules with and without elastography. Eur. J. Endocrinol..

[19] Garino F., Deandrea M., Motta M., Mormile A., Ragazzoni F., Palestini N., Freddi M., Gasparri G., Sgotto E., Pacchioni D. (2015). Diagnostic performance of elastography in cytologically intermediate thyroid nodules. Endocrine.

[20] Habib L.A.M., Abdrabou A.M., Geneidi E.A.S., Sultan Y.M. (2016). Role of ultrasound elastography in assessment of indeterminate thyroid nodules. Egypt. J. Radiol. Nucl. Med..

[21] Lloyd R.V., Osamura R.Y., Kloppel G., Rosai J. (2017). WHO Classification of Tumours of Endocrine Organs.

[22] Kakudo K. (2018). How to handle borderline/precursor thyroid tumors in management of patients with thyroid nodules. Gland Surg..

[23] Bychkov A., Hirokawa M., Jung C.K., Liu Z., Zhu Y., Hong S.W., Satoh S., Lai C.R., Huynh L., Kakudo K. (2017). Low rate of noninvasive follicular thyroid neoplasm with papillary like nuclear features in Asian practice. Thyroid.

[24] Liu Z., Song Y., Han B., Zhang X., Su P., Cui X. (2017). Non-invasive follicular thyroid neoplasm with papillary-like nuclear features and the practice in Qilu Hospital of Shandong University, China. J. Basic Clin. Med..

[25] Jung C.K., Kim C. (2017). Effect of lowering the diagnostic threshold for encapsulated follicular variant of papillary thyroid carcinoma on the prevalence of non-invasive follicular thyroid neoplasm with papillary-like nuclear features: A single-institution experience in Korea. J. Basic Clin. Med..

[26] Baloch Z.W., Fleisher S., LiVolsi V.A., Gupta P.K. (2002). Diagnosis of “follicular neoplasm”: A gray zone in thyroid fine-needle aspiration cytology. Diagn. Cytopathol..

[27] Rago T., Di Coscio G., Basolo F., Scutari M., Elisei R., Berti P., Miccoli P., Romani R., Faviana P., Pinchera A. (2007). Combined clinical, thyroid ultrasound and cytological features help to predict thyroid malignancy in follicular and Hupsilonrthle cell thyroid lesions: Results from a series of 505 consecutive patients. Clin. Endocrinol..

[28] Rago T., Scutari M., Santini F., Loiacono V., Piaggi P., Di Coscio G., Basolo F., Berti P., Pinchera A., Vitti P. (2010). Real-time elastosonography: Useful tool for refining the presurgical diagnosis in thyroid nodules with indeterminate or nondiagnostic cytology. J. Clin. Endocrinol. Metab..

[29] Sorrenti S., Trimboli P., Catania A., Ulisse S., De Antoni E., D’Armiento M. (2009). Comparison of malignancy rate in thyroid nodules with cytology of indeterminate follicular or indeterminate Hürthle cell neoplasm. Thyroid.

[30] Mufti S.T., Molah R. (2012). The bethesda system for reporting thyroid cytopathology: A five-year retrospective review of one center experience. Int. J. Health Sci..

[31] Park J.H., Yoon S.O., Son E.J., Kim H.M., Nahm J.H., Hong S. (2014). Incidence and malignancy rates of diagnoses in the bethesda system for reporting thyroid aspiration cytology: An institutional experience. Korean J. Pathol..

[32] Trimboli P., Guglielmi R., Monti S., Misischi I., Graziano F., Nasrollah N., Amendola S., Morgante S.N., Deiana M.G., Valabrega S. (2012). Ultrasound sensitivity for thyroid malignancy is increased by real-time elastography: A prospective multicenter study. J. Clin. Endocrinol. Metab..

[33] Lippolis P.V., Tognini S., Materazzi G., Polini A., Mancini R., Ambrosini C.E., Dardano A., Basolo F., Seccia M., Miccoli P. (2011). Is elastography actually useful in the presurgical selection of thyroid nodules with indeterminate cytology?. J. Clin. Endocrinol. Metab..

[34] Esfahanian F., Aryan A., Ghajarzadeh M., Yazdi M.H., Nobakht N., Burchi M. (2016). Application of sonoelastography in differential diagnosis of benign and malignant thyroid nodules. Int. J. Prev. Med..

[35] Schenke S., Zimny M. (2018). Combination of Sonoelastography and TIRADS for the Diagnostic Assessment of Thyroid Nodules. Ultrasound Med. Biol..

[36] Ragazzoni F., Deandrea M., Mormile A., Ramunni M.J., Garino F., Magliona G., Motta M., Torchio B., Garberoglio R., Limone P. (2012). High diagnostic accuracy and interobserver reliability of real-time elastography in the evaluation of thyroid nodules. Ultrasound Med. Biol..

[37] Vorländer C., Wolff J., Saalabian S., Lienenlüke R.H., Wahl R.A. (2010). Real-time ultrasound elastography—a noninvasive diagnostic procedure for evaluating dominant thyroid nodules. Langenbecks Arch. Surg..

[38] Friedrich-Rust M., Sperber A., Holzer K., Diener J., Grünwald F., Badenhoop K., Weber S., Kriener S., Herrmann E., Bechstein W.O. (2010). Real-time elastography and contrast-enhanced ultrasound for the assessment of thyroid nodules. Exp. Clin. Endocrinol. Diabetes.

[39] Xue J., Cao X.L., Shi L., Lin C.H., Wang J., Wang L. (2016). The diagnostic value of combination of TI-RADS and ultrasound elastography in the differentiation of benign and malignant thyroid nodules. Clin. Imaging.

[40] Rago T., Santini F., Scutari M., Pinchera A., Vitti P. (2007). Elastography: New developments in ultrasound for predicting malignancy in thyroid nodules. J. Clin. Endocrinol. Metab..

[41] Miller A.B., Chamberlain J., Day N.E., Hakama M., Prorok P.C. (1990). Report on a workshop of the UICC project on evaluation of screening for cancer. Int. J. Cancer..

